# The clinical meaning of the area under a receiver operating characteristic curve for the evaluation of the performance of disease markers

**DOI:** 10.4178/epih.e2022088

**Published:** 2022-10-17

**Authors:** Stefano Parodi, Damiano Verda, Francesca Bagnasco, Marco Muselli

**Affiliations:** 1 Scientific Directorate, IRCCS Istituto Giannina Gaslini, Genoa, Italy; 2 Rulex Innovation Labs, Genoa, Italy; 3 Institute of Electronics, Computer and Telecommunication Engineering, National Research Council of Italy, Genoa, Italy

**Keywords:** Receiver operating characteristic curve, Biomarkers, Diagnostic accuracy, Binormal model, Metz and Kronman test

## Abstract

**OBJECTIVES:**

The area under a receiver operating characteristic (ROC) curve (AUC) is a popular measure of pure diagnostic accuracy that is independent from the proportion of diseased subjects in the analysed sample. However, its actual usefulness in the clinical context has been questioned, because it does not seem to be directly related to the actual performance of a diagnostic marker in identifying diseased and non-diseased subjects in real clinical settings. This study evaluates the relationship between the AUC and the proportion of correct classifications (global diagnostic accuracy, GDA) in relation to the shape of the corresponding ROC curves.

**METHODS:**

We demonstrate that AUC represents an upward-biased measure of GDA at an optimal accuracy cut-off for balanced groups. The magnitude of bias depends on the shape of the ROC plot and on the proportion of diseased and non-diseased subjects. In proper curves, the bias is independent from the diseased/non-diseased ratio and can be easily estimated and removed. Moreover, a comparison between 2 partial AUCs can be replaced by a more powerful test for the corresponding whole AUCs.

**RESULTS:**

Applications to 3 real datasets are provided: a marker for a hormone deficit in children, 2 tumour markers for malignant mesothelioma, and 2 gene expression profiles in ovarian cancer patients.

**CONCLUSIONS:**

The AUC is a measure of accuracy with potential clinical relevance for the evaluation of disease markers. The clinical meaning of ROC parameters should always be evaluated with an analysis of the shape of the corresponding ROC curve.

## INTRODUCTION

Receiver operating characteristic (ROC) curves are standard statistical tools for the analysis of disease markers (DMs). The area under a ROC curve (AUC) is a popular measure of diagnostic accuracy that represents an estimator of the probability of correctly ranking a pair of subjects: one extracted from the class of diseased individuals and the other from that of non-diseased individuals [1].

Despite its popularity, the actual usefulness and the meaning of the AUC in clinical settings have been intensely questioned. One of the major criticisms is that patients are not presented to physicians in diseased/non-diseased pairs; thus, the clinical meaning of the AUC remains unclear [2–5]. Furthermore, the AUC is an average measure of sensitivity calculated over the complete set of specificity values, including those with poor clinical relevance. For this reason, in many instances the partial area (pAUC) is preferred to the AUC. The pAUC can be computed in a range of specificity or sensitivity values that are deemed meaningful [6,7]. In most cases, the pAUC is calculated between 0 and the highest acceptable false positive rate, and the performance of 2 DMs is evaluated by comparing the 2 corresponding pAUCs [3,8,9]. However, such a comparison may be prone to insufficient statistical power, the results of the test can strongly depend on the selected range of specificity, and the estimation of the pAUC is likely to be more noticeably affected than that of the AUC by the shape of the fitted ROC curve [10,11].

In the next paragraphs, we briefly review the principles of ROC analysis. We show that the AUC represents an optimistic (i.e., upward-biased) estimate of the proportion of subjects that could be correctly classified by a binary test in the presence of balanced groups (the same proportion of diseased and non-diseased patients). We demonstrate that the relationship between AUC and global accuracy is independent from the proportion of diseased subjects when the curve has a regular shape (a proper ROC curve). Finally, we demonstrate that, under a proper model, 2 pAUCs can be compared by a more powerful test for the corresponding AUCs. For illustration, we present applications to 3 real datasets for the evaluation of the diagnostic accuracy of different DMs.

The R scripts [12] developed *ad hoc* for the present study are listed in the Supplementary Material 1 with an example of their application.

## MATERIALS AND METHODS

### Principles of receiver operating characteristic analysis

The early steps for marker validation, in general, require a case-control approach; therefore, following a widespread convention, in this article the class of diseased subjects will be referred to as “cases” and the referent group as “controls” [13].

Given a continuous DM, a binary diagnostic test can be defined selecting a cut-off value *c* and classifying as test-positive any DM value >*c*. The probability of a correct classification among cases is called sensitivity, *Se(c)* (also referred to as the true-positive rate), and that among controls is referred to as specificity, *Sp(c)* (the false-positive rate). An empirical ROC curve is obtained by plotting each *Se(c)* vs. the corresponding 1-*Sp(c)* for any observed *c* [8].

The global diagnostic accuracy (GDA) represents the probability of a correct classification at each cut-off *c* and is a linear function of *Se(c)* and *Sp(c)* [14]:


(1) GDA(c)=Se(c)·π+Sp(c)·(1-π)

where π represents the proportion of cases (often referred to as “disease prevalence”).

Combining *Se(c)* and *Sp(c)*, 2 measures of accuracy independent from π can be obtained (pure accuracy indices), namely, the Youden index *(J)*:


(2) J(c)=Se(c)+Sp(c)-1

and the diagnostic odds ratio (DOR) [14]:


(3) $$DOR(c)=\frac{Se(c)}{1-Sp(c)}\cdot \frac{Sp(c)}{1-Se(c)}$$

*J* corresponds to the vertical distance of the ROC curve from the rising diagonal. Its maximum value, which equals the Kolmogorov-Smirnov test statistic, identifies an “optimal” cut-off that corresponds to the minimum value of the sum of false positive and false negative proportions [8].

Replacing *Se(c)* with y and 1-*Sp(c)* with *x* in equation (3), the equation of a ROC curve is obtained as a function of DOR; in the case of a constant DOR, it becomes [15]:


(4) $$y=\frac{DOR\cdot x}{DOR\cdot x+1-x}$$

**Figure 1:**
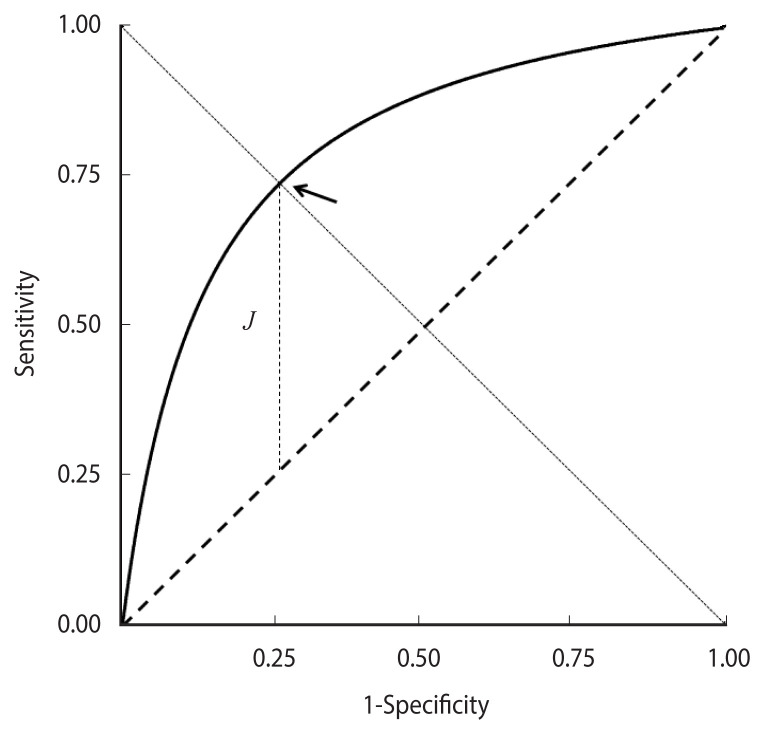
A theoretical proper receiver operating characteristic curve. The cut-off corresponding to the maximum value of the Youden index *J* is indicated by an arrow.

Equation (4) can also be obtained from a “proper” binormal model—that is, hypothesising that DM values follow a normal distribution with different means in cases and controls and equal variances. Accordingly, the corresponding graph is called a proper binormal ROC curve [15–18]. The curve is concave and symmetric with respect to the descending diagonal and the point of the highest Youden index *J* corresponds to equal values of *Se(c)* and *Sp(c)* (Figure 1).

An empirical ROC curve is invariant with respect to any transformation of the corresponding DM values that does not alter their original ranks; then, in a very general framework, the standard binormal model can be assumed if the DM distribution in cases and controls is transformable into 2 normal distributions by a monotonic function [19]. The correspondence between equation (4) and the parametric binormal model allows the application of statistical methods to assess the shape of a ROC curve. For instance, the departure of an empirical ROC curve from the proper model can be evaluated by the test proposed by Metz & Kronman [18].

### The clinical meaning of the area under a receiver operating characteristic curve

#### The AUC is an estimator of GDA that depends on the shape of the ROC curve

**Figure 2:**
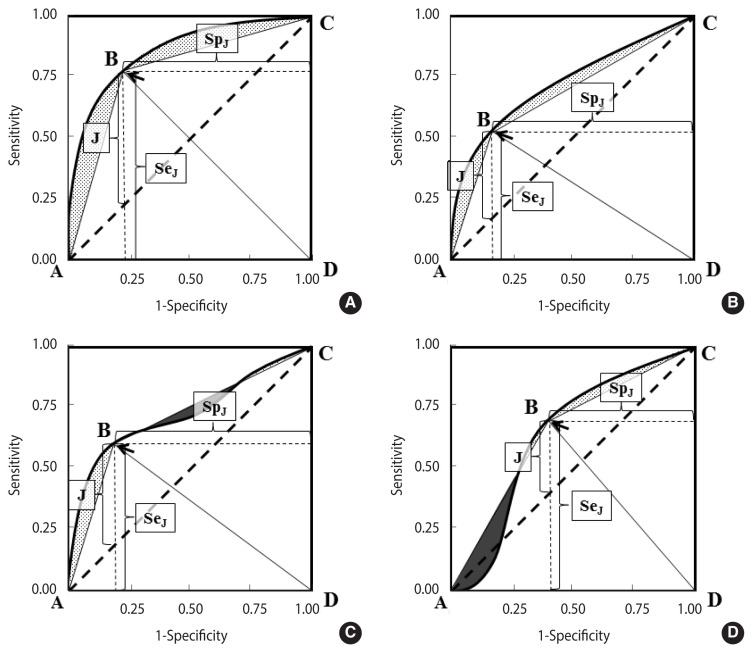
Association between AUC and GDA (area of the ABCD polygon) in 1 proper binormal and 3 differently shaped ROC curves. (A) Proper binormal ROC curve, (B) concave and asymmetric ROC curve, (C) non-proper ROC curve, non-concave and not crossing the chance line, and (D) non-proper ROC curve, non-concave and crossing the line of chance (a “wiggly” ROC curve). The area under the ROC curve and above the ABCD polygon is coloured grey, while the area above the ROC curve and under the polygon (observed in non-concave ROC plots, panels C and D) is depicted in black. ROC, receiver operating characteristic; AUC, area under the ROC curve; GDA, global diagnostic accuracy.

Figure 2A–D shows 4 differently shaped ROC curves.

In each curve, the optimal cut-off, corresponding to the highest Youden index (equation [2]), is indicated by an arrow.

A polygon ABCD can be obtained by joining a point to the origin and to the upper right corner of the ROC plot. It can be noted that the area A of ABCD is a biased measure of the AUC:


AUC=A(ABCD)+b

where *b* represents the “bias” that in concave curves corresponds to the area between the curve and the polygon (Figure 1, grey regions in panels A and B).

The area delimited by ABCD is the sum of the areas under the 2 triangles ABD and BCD, whose bases are both equal to one and the heights are, respectively, the sensitivity and the specificity at the optimal cut-off:


(5) $$\text{A}(ABCD)=\frac{1}{2}Se_{J}+\frac{1}{2}Sp_{J}$$

It can be noted that equation (5) is equivalent to equation (1) in the presence of a balanced sample (π=0.5), then:


A(ABCD)=GDA(π=0.5)

In a proper binormal theoretical ROC curve (Figure 2A), the optimal cut-off corresponds to the point of equal sensitivity and specificity; then, replacing *Sp_J_* with *Se_J_* in equation (5), it is easy to verify that *GDA* no longer depends on π:


(6) $$\text{A}(ABCD)=GDA_{J}=Se_{J}$$

In summary, the AUC represents an “optimistic” estimator of GDA; that is, it provides an upper bound for the proportion of correct classifications that can be obtained at an optimal accuracy cut-off and in the presence of 2 balanced groups. An interesting property of the proper binormal ROC curves is that the association between the AUC and GDA at the optimal cut-off is independent from the proportion of cases and controls.

#### Relationship between b and DOR

The amount of bias, *b*, depends on the shape of the ROC curve. A loss of concavity can reduce *b*, in part or completely (Figure 2, dark regions in panels C and D). Conversely, under a proper model (Figure 2A), *b* tends to be higher, but it can be calculated by applying the following equation:


(7) $$b=\frac{\left(DOR-1)\cdot \sqrt{DOR}-DOR\cdot ln(DOR\right)}{{\left(DOR-1\right)}^{2}}$$

as demonstrated in the Appendix 1. The DOR can be calculated from the corresponding AUC, using the following equation, obtained by integrating equation (4) [20]:


(8) $$AUC=\frac{DOR}{DOR-1}\cdot \left(1-\frac{ln(DOR)}{DOR-1}\right)$$

A routine in R language (*rocdor*) is provided in the Supplementary Material 1 to calculate the DOR from the AUC via a numeric approach. The confidence intervals of the DOR can be estimated from equation (8) by replacing the AUC with the corresponding confidence intervals, obtained using the method of DeLong et al. [21].

**Figure 3:**
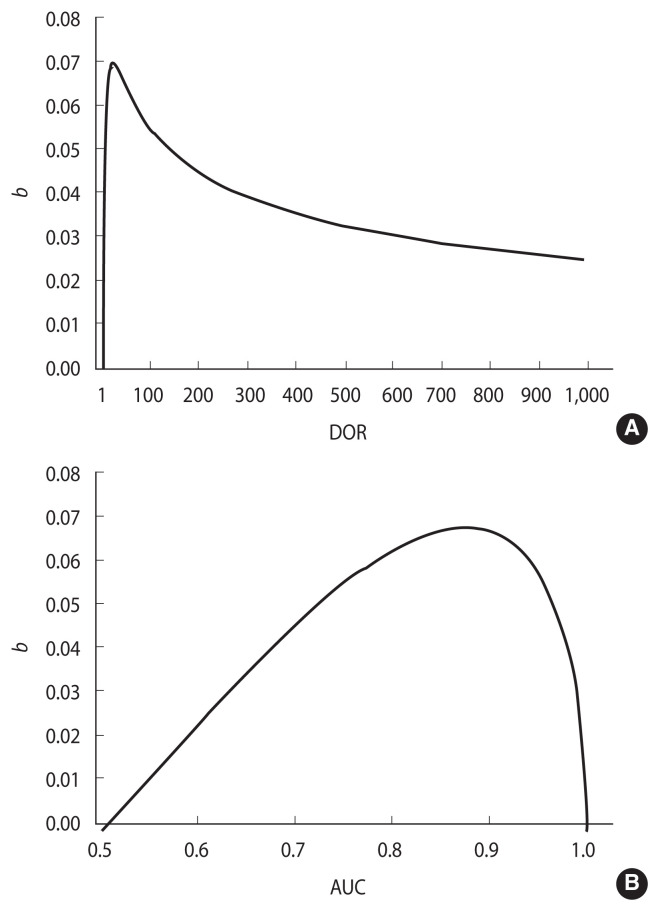
Relationship of the difference between the AUC and GDA (“bias” b) at the optimal cut-off in a proper ROC curve as a function of DOR (A) and AUC (B). ROC, receiver operating characteristic; AUC, area under the ROC curve; GDA, global diagnostic accuracy; DOR, diagnostic odds ratio.

Figure 3 shows the relationship between *b* and the DOR, according to equation (7) (Figure 3A) and between *b* and the AUC (Figure 3B). The highest *b* value (0.07) is observed for DOR=17.3, which corresponds to an AUC of 0.876. *b* tends toward 0 when the AUC is close to either 0.5 or 1.0. The first case corresponds to a DOR of 1.0 and to a non-informative ROC curve lying on the rising diagonal, while the latter corresponds to an infinite DOR and a perfect separation between DM values in cases and controls (Figure 3).

#### Relationship between the AUC, pAUC and DOR

Integrating equation (4) from 0 to *k*, *k*≤1, the association between the DOR and the partial area pAUC(*k*) between a specificity of *k* and 1.0 is obtained [22]:


(9) $$pAUC(k)=k\cdot \frac{DOR}{DOR-1}\left(1-\frac{ln(1+k\cdot (DOR-1))}{k\cdot (DOR-1)}\right)$$

**Figure 4:**
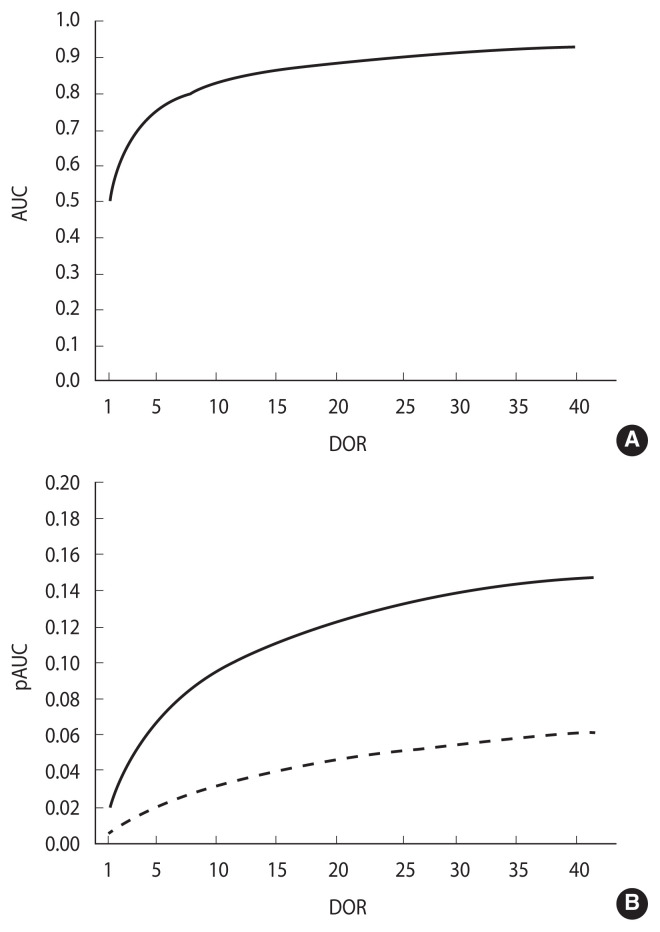
The AUC and partial AUC (pAUC) as functions of DOR in a proper ROC model. (A) AUC, (B) pAUC between 0.00 and 0.80 specificity values (solid line) and between 0.00 and 0.90 specificity values (dashed line). ROC, receiver operating characteristic; AUC, area under the ROC curve; DOR, diagnostic odds ratio.

In a proper model, the association of both AUC and pAUC(*k*) with the DOR (equations (8) and (9), respectively) is monotonic and strictly increasing in the DOR, as depicted in Figure 4 (panels A and B, respectively). Accordingly, the inequalities AUC_1_>AUC_2_ and pAUC_1_>pAUC_2_ both imply and are implied by DOR_1_>DOR_2_. As a consequence, under a proper model assumption, the difference between 2 pAUCs can be assessed by testing the difference between the corresponding whole AUCs (Figure 4).

#### Comparison between 2 ROC curves

The departure from a proper model of 2 empirical ROC curves can be checked by the Metz and Kronman test [18]. In the case of a significant result, a usual test for the comparison of 2 pAUCs can be employed. In this study, the bootstrap method by Robin et al. [9] was adopted and implemented by the authors in the pROC R library. Conversely, if the hypothesis of a proper model is not rejected, a standard test for the comparison of the corresponding AUCs is suggested to improve statistical power. In this investigation, we employed the method proposed by DeLong et al. [21].

### Data sets selected for the application to real data in diagnostic medical settings

The first dataset (“GH deficit”) included 79 patients with a reversible growth hormone deficit (GHD) during childhood, as diagnosed by the insulin-tolerance test (ITT). The ITT measured at first diagnosis was eventually evaluated as a DM for permanent GHD during late adolescence. Permanent GHD was observed in 31 out of the 79 recruited patients [23].

The second dataset (“malignant mesothelioma”) included 52 patients with malignant pleural mesothelioma and 117 patients affected by either benign pleurisies (n=55) or pleural metastases from other malignancies (n=62), and 2 tumour markers (namely, SMRP and Cyfra21-1) [24].

The third dataset (“ovarian cancer”) included the expression of more than 1,500 genes in normal (n=23) and malignant (n=30) ovarian tissues [8]. In this study only the first 2 genes were analysed.

The results of the application of the proposed approach of analysis are resumed in Supplementary Material 2 and illustrated in detail in the paragraphs below.

### Ethics statement

Neither ethical committee approval nor informed consent were needed because the data analysed are publicly available on previously published papers and they are completely anonymized.

## RESULTS

### Area under the receiver operating characteristic (ROC) curve is an estimator of the global diagnostic accuracy at an optimal cut-off in a proper ROC curve

**Figure 5:**
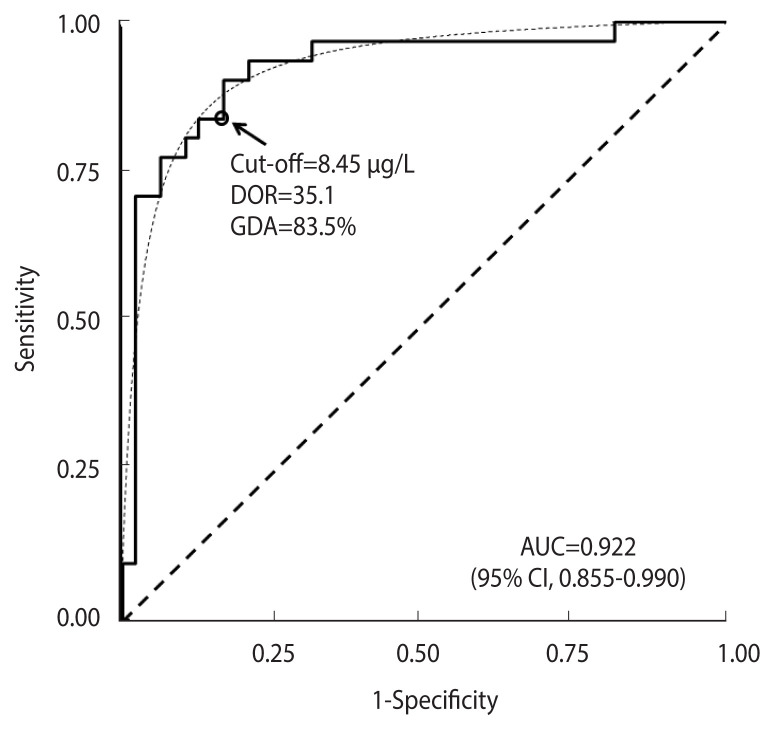
Receiver operating characteristic (ROC) curve for the ITT test in 31 cases with permanent GH deficit and 48 healthy controls. The corresponding theoretical proper curve is displayed as a dashed line. The area under the ROC curve (AUC) with a 95% confidence interval (CI), the cut-off value corresponding to equal values of sensitivity and specificity (indicated by a circle), the diagnostic odds ratio (DOR), and the corresponding global diagnostic accuracy (GDA) are shown. ITT, insulin-tolerance test; GH, growth hormone.

Figure 5 shows the ROC curve obtained from the ITT test in the “GH deficit” dataset. The empirical estimate of AUC was 0.922 (95% CI, 0.855 to 0.990). The ROC curve fit the expected theoretical proper ROC very well (p=0.644, Metz and Kronman test). The corresponding DOR was 35.1. The cut-off of equal values for sensitivity and specificity corresponded to a DM value of 8.45 μg/L, as is indicated by a circle in Figure 5. The corresponding observed GDA was 83.5%.

Applying equation (7), an estimate of the bias *b* of 0.066 (95% CI, 0.032 to 0.069) was obtained, which, subtracted from $\hat{\text{AUC}}$, provided an estimate of GDA of 86% (95% CI, 79 to 96), very close to the observed value (Figure 5 and Supplementary Material 2).

### Comparison between a proper and a non-proper receiver operating characteristic curve

**Figure 6:**
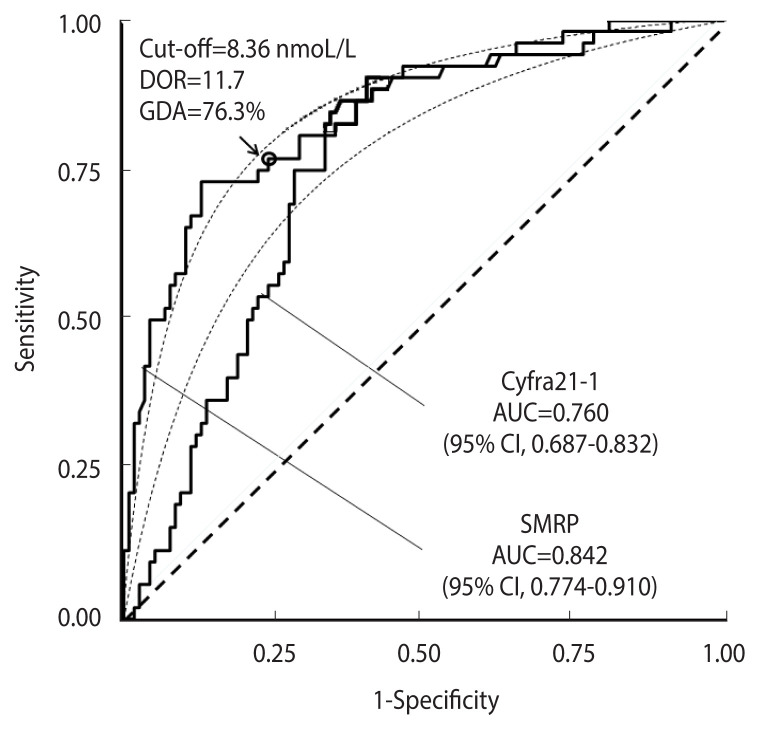
Receiver operating characteristic (ROC) curves for the SMRP and CYFRA21-1 tumour markers in 52 cases and 117 controls from the “malignant mesothelioma” dataset. The corresponding theoretical proper curves are shown as dashed lines. The areas under the ROC curves (AUCs) with 95% confidence intervals (CIs) are reported. The cut-off corresponding to equal values of sensitivity and specificity (indicated by a circle), the diagnostic odds ratio (DOR), and the corresponding global diagnostic accuracy (GDA) are shown for the SMRP curve that is consistent with the proper model.

Figure 6 shows a comparison between ROC curves corresponding to the distribution of the SMRP and Cyfra21-1 tumour markers in the “malignant mesothelioma” dataset.

The AUC for SMRP was 0.842 (95% CI, 0.774 to 0.910), and that for Cyfra21-1 was 0.760 (95% CI, 0.687 to 0.832). The cut-off corresponding to equal values of sensitivity and specificity, as well as the DOR and GDA estimates, are shown in Figure 6, while the corresponding bias *b*, obtained according to equation (8), and the expected GDA are reported in Supplementary Material 2. With regard to Cyfra21-1, the corresponding ROC curve (Figure 6) was clearly asymmetrical, with a statistically significant departure from the theoretical proper model (p=0.003, Metz and Kronman test). Thus, to compare the 2 ROC curves, a test for the comparison between 2 pAUCs could be more appropriate than a standard test for the corresponding whole AUCs. For instance, in the specificity range of 0.80 to 1.00, the difference between the 2 corresponding pAUCs was 0.070 and the corresponding p-value was highly statistically significant (p<0.001). Applying a standard test for the comparison of the AUCs, the difference between the 2 corresponding whole AUCs was only marginally statistically significant (p=0.043) (Figure 6).

### Comparison between 2 proper receiver operating characteristic curves

**Figure 7:**
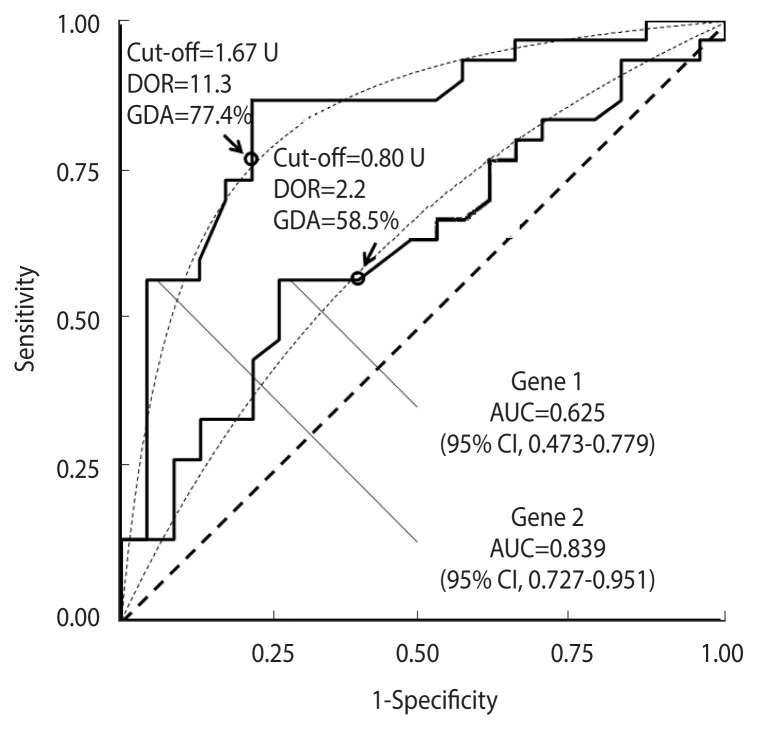
Receiver operating characteristic (ROC) curves for the expression of gene 1 and gene 2 in the “ovarian cancer” database (30 cases and 23 controls). The corresponding theoretical proper curves are shown as dashed lines. The area under the ROC curve (AUC) with a 95% confidence interval (CI), the cut-off corresponding to equal values of sensitivity and specificity (indicated by a circle), the diagnostic odds ratio (DOR), and the corresponding global diagnostic accuracy (GDA) are shown.

Figure 7 shows a comparison between the ROC curves corresponding to the expression of the first 2 genes in the “ovarian cancer” dataset.

The AUC for gene 1 was 0.625 (95% CI, 0.473 to 0.779), and that for gene 2 was 0.839 (95% CI, 0.727 to 0.951). The cut-offs corresponding to equal values of sensitivity and specificity, and the DOR and GDA estimates are also displayed. The corresponding bias *b*, estimated according to equation (8), and the expected GDA are reported in Supplementary Material 2.

Both empirical curves fitted the theoretical plot quite well (p=0.546 for gene 1 and p=0.645 for gene 2, respectively), indicating that the comparison of the 2 whole AUCs was appropriate. The test result (p=0.027) suggested that the performance of gene 2 could be higher than that of gene 1 when used as a DM for the diagnosis of ovarian cancer. Conversely, if a test for the 2 corresponding pAUCs in the specificity range of 0.80–1.00 had been applied, a p-value of 0.141 would have been obtained (Figure 7).

## DISCUSSION

Since its first application in clinical epidemiology in the early 1980s [25], ROC analysis has become an increasingly popular method to assess the performance of DMs, which in combination with patients’ clinical and demographic characteristics provides valid support for the differential diagnosis of several diseases [5]. However, the use of the AUC for diagnostic applications has often been criticised for many reasons, including the fact that the connection between the AUC and the probability of a correct classification remains unclear [4,5]. We have demonstrated that the AUC represents an upper limit of the expected global accuracy at an optimal cut-off and for balanced groups. In proper curves, the actual GDA at an optimal cut-off can be obtained from the AUC, and it is independent from the proportion of cases in the studied sample. The latter property may have important clinical consequences, because the accuracy of a DM at an optimal cut-off can be estimated using a suitable sampling ratio, provided that cases and controls are representative of the general population of diseased and non-diseased individuals, respectively. However, the choice of a suitable cut-off depends on the costs associated with false-positive and false-negative results and the expected proportion of diseased subjects in the target population [26]. In this case, the clinical usefulness of AUC remains to be clarified.

An interesting property of proper ROC curves is that the comparison between 2 pAUCs can be performed by a test for 2 AUCs with a strong improvement of the statistical power. We applied the Metz and Kronman test [18] to distinguish between proper and non-proper ROC curves, which could guide the choice of the most suitable approach for the comparison of 2 curves.

The results of the present investigation strongly support the statement by Janssens & Martens [5] that AUC should always be interpreted considering the shape of the entire ROC curve, which mirrors the performance of the underlying marker at different ranges of values. A proper model implicitly assumes that the disease is associated with an increase in DM values that is homogeneous within the group of cases [8]. In the presence of strong departures from the proper model, the AUC no longer provides a useful measure of the potential performance of the underlining diagnostic marker, and some new ROC-based parameters have recently been proposed, including the area under a generalised ROC curve [27] and the length of a ROC curve [28]. Moreover, a departure from the assumption of a proper curve may reflect the presence of heterogeneous subgroups among either group under study. For example, wiggly ROC curves may indicate the presence of hidden subgroups inside either analysed group [8,29–31], while asymmetric curves that do not cross the line of chance may indicate that only a subgroup of cases differs from controls for the presence of higher DM values [32,33]. Finally, the loss of concavity in empirical curves can be quite common even if the corresponding theoretical ROC curve is concave [16]. In such a case, the corresponding AUC estimate can be affected by a negligible bias, whereas the pAUC is more likely to be overestimated or underestimated [16]. Further investigations are needed to develop new methods to identify concave non-symmetric ROC curves, and to elucidate the relationship between the AUC and GDA.

The results of this study should be considered in light of some limitations. ROC analysis has been criticised when applied to analyse risk score profiles in that a clinically significant change in the probability of disease for 1 or more subjects (the risk estimate) might not be reflected by an appreciable increase in the corresponding AUC [5,34]. Furthermore, the usefulness of ROC analysis for assessing the improvement in prediction performance gained by adding a DM to a baseline predictor remains controversial, even though some alternatives recently proposed have been criticised as well [35].

In conclusion, our study indicates that the AUC is a useful measure of accuracy with potential clinical relevance for the evaluation of DMs and that it represents an upper limit of disease accuracy when the curve shape is consistent with the binomial proper model. In this case, the GDA at an optimal cut-off is independent from the sampling ratio, thus favouring the design of suitable studies for the evaluation of potential diagnostic markers. Finally, for binormal proper curves, a statistical comparison between 2 or more pAUCs is equivalent to the comparison of the corresponding whole AUCs, which has a higher statistical power. ROC parameters, including the AUC and the pAUC, should always be evaluated in combination with an analysis of the shape of the corresponding ROC curve.

## Appendix 1 Demonstration of equation (7)

Given a proper binormal ROC curve, let *S**_eJ_* = *S**_pJ_* be the sensitivity corresponding to the optimal cut-off. Applying equation (3), the following relationship between DOR and *S**_eJ_* is obtained:

$$DOR=\frac{Se_{J}}{1-Sp_{J}}\cdot \frac{Sp_{J}}{1-Se_{J}}=\frac{Se_{J}^{2}}{{\left(1-Se_{J}\right)}^{2}}$$

whose solutions in *S**_eJ_* are:

$$Se_{J}=\frac{DOR\mp \sqrt{DOR}}{DOR-1}$$

The highest value must be rejected because for any DOR >1, a sensitivity value >1 would be obtained. Applying equation (6) to the lowest solution:

$$\text{A}(ABCD)=\frac{DOR-\sqrt{DOR}}{DOR-1}$$

The bias *b* can now be estimated by subtracting A(*ABCD)* from the AUC and by applying equation (8):

$$b=AUC-\text{A}(ABCD)=\frac{DOR}{DOR-1}\cdot (1-\frac{ln(DOR)}{DOR-1})-\frac{DOR-\sqrt{DOR}}{DOR-1}=\frac{\left(DOR-1)\cdot \sqrt{DOR}-DOR\cdot ln(DOR\right)}{{\left(DOR-1\right)}^{2}}$$
