# Supplementary material for: The clinical meaning of the area under a receiver operating characteristic curve for the evaluation of the performance of disease markers
Source: Epidemiol Health. 2022 Oct 17;44:e2022088. doi: 10.4178/epih.e2022088 (PMC10089712; doi:10.4178/epih.e2022088)
Supplement: Supplementary file 1 [file epih-44-e2022088-Supplementary-1.docx]

SUPPLEMENTAL MATERIAL

Supplementary Material 1. Results of the application of the proposed approach of ROC analysis to five potential diagnostic markers from three real data sets.

| Disease Marker | AUC | *95%CI* | DOR | *b* | GDA_se_ | Exp.  GDA | MKT  *p*-value |
| --- | --- | --- | --- | --- | --- | --- | --- |
| ITT Test (Figure 5) | 0.922 | *0.855 – 0.990* | 35.1 | *0.0663* | 83.5% | 85.6% | 0.644 |
| Cyfra21-1 (Figure 6) | 0.760 | *0.687 – 0.832* | *n.e.* | *n.e.* | 71.6% | *n.e.* | **0.003** |
| SMRP (Figure 6) | 0.842 | *0.774 – 0.910* | 11.7 | *0.0683* | 76.3% | 77.4% | 0.348 |
| Gene 1 (Figure 7) | 0.625 | *0.473 – 0.779* | 2.2 | *0.0306* | 58.5% | 59.4% | 0.546 |
| Gene 2 (Figure 7) | 0.839 | *0.727 – 0.951* | 11.3 | *0.0681* | 77.4% | 77.1% | 0.644 |

AUC = Area Under the ROC Curve; 95%CI = 95% Confidence Interval of AUC; DOR = Diagnostic Odds Ratio, estimated applying equation 8; *b* = “bias” estimated applying equation 7; GDA_se_ = Global Diagnostic Accuracy corresponding to the point of equal sensitivity and specificity on the ROC curve; Exp. GDA = Expected Global Diagnostic Accuracy calculated by subtracting *b* from AUC; MKT= Metz and Kronman test to evaluate the departure of an empirical ROC curve from the theoretical proper model; *n.e.*= not evaluable, because the corresponding ROC curve is not proper.
