# Supplementary material for: The clinical meaning of the area under a receiver operating characteristic curve for the evaluation of the performance of disease markers
Source: Epidemiol Health. 2022 Oct 17;44:e2022088. doi: 10.4178/epih.e2022088 (PMC10089712; doi:10.4178/epih.e2022088)
Supplement: Supplementary file 2 [file epih-44-e2022088-Supplementary-2.docx]

SUPPLEMENTAL MATERIAL

Supplementary Material 2. Routines in R language

The following R scripts include the application of the Metz and Kronman test for the evaluation of a proper binormal ROC curve, implemented in the *MKT*() function. Before using *MKT()*, the corresponding parameters have to be estimated by the *rocreg*() function included in the “pcvsuite” library, developed in R language by Bansal A, Morris DM, Longton G, Pepe MS and Janes H, and available at the following web site: *http://faculty.washington.edu/abansal/software.html*. The other R functions listed below include routines to estimate: a) the ROC curve coordinates (function *roctable()*); b) the area under an empirical ROC curve (*rocauc()*) and the corresponding standard error and 95%CI (*rocseauc()*) by the DeLong *et al* method (1988); c) the *AUC* corresponding to a specified *DOR* value in a proper binormal curve (*rocaucfromdor()*) according to equation (9); d) the *DOR* from an *AUC* value under a binormal proper model assumption (*rocdor()*), applying a “divide-and-conquer” algorithm to equation (9); e) the “bias” in equation (8) (*biasproproc()*). Furthermore, a simple graphical function to plot one or more empirical ROC curves is provided (*plotroc()*) together with the commands to add a theoretical proper binormal curve to the ROC plot (*plotproper()*). Finally, a routine to obtain the coordinates of a theoretical binormal proper curve according to equation (4) is provided (function *proproc()*).

As an example of application of the R scripts reported below, the following commands reproduce the main results of the application of the proposed method to the ITT data set, illustrated in the paragraph 6.2. Some output, not relevant for illustrative purposes, has been omitted. The data set used in the example (ITTData), as well the other databases cited in the manuscript, is available as Supplementary Material file.

######################################################################

###### R SCRIPTS USED IN THE PRESENT STUDY ######

######################################################################

MKT <- function(ROCmatrix, ROCParm) {

# Metz and Kronmam test for binormal proper ROC curves

# ROCmatrix: ROC matrix generated by roctable() function

#

# ROCParm: parameters generated by rocreg() (pcvsuite library)

# ROCparm is generated by using the following syntax:

# library(pcvsuite)

# ROC_Parm <- rocreg(dataset = "<dataset name>", d = "<status variable name>,

# markers = "<marker variable name>")

#

# Parameters for the Metz and Kronman test:

# a_e: expected value of a (obtained from AUC)

# b_e: expected value of b (it must be 1 for proper ROC curves)

# a_o: observed value of a (estimated by rocreg())

# b_o: observed value of b (estimated by rocreg())

# a_v: variance of a (estimated by rocreg())

# b_v: variance of b (estimated by rocreg())

# ab_cov: a,b covariance (estimated by rocreg())

# ab_cor: correlation between a and b (calculated using a_v, b_v and ab_cov)

a_e <- qnorm(rocauc(ROCmatrix))*sqrt(2)

b_e <- 1.0

a_o <- ROCParm$GLMparm[1]

b_o <- ROCParm$GLMparm[2]

a_v <- ROCParm$rocreg_m1$V[1,1]

b_v <- ROCParm$rocreg_m1$V[2,2]

ab_cov <- ROCParm$rocreg_m1$V[2,1]

ab_cor <- ab_cov/(sqrt(a_v) * sqrt(b_v))

chival <- 1/(1-ab_cor^2)* ((a_o - a_e)^2 / a_v + (b_o - b_e)^2 / b_v +

2*ab_cor*(a_o-a_e)*(b_o-b_e)/(sqrt(a_v) * sqrt(b_v)))

pval <- 1-pchisq(chival,2)

return(pval)

}

roctable <- function(marker, status) {

# Estimate of the coordinates of the ROC curve

# Making a sorted data frame without missing values

data <- data.frame(Marker = marker, Status = status)

data <- na.omit(data)

data <- data[order(data$Marker),]

# Initializing the ROC matrix

nsam <- length(data$Status)

ncases <- sum(data$Status)

ncontr <- nsam - ncases

rocmatr <- matrix(nrow=nsam+1, ncol= 3)

rocmatr[1,1] <- 1 # First value of sensitivity

rocmatr[1,2] <- 1 # First value of 1 - specificity

rocmatr[1,3] <- data$Marker[1] # First cut-off

colnames(rocmatr) <- c("Sens", "1-Spec", "Cut-off")

# Calculating the ROC curve coordinates

for (i in 1:nsam) {

sens <- spec <- 0.0

for (j in 1:nsam) {

if ((data$Marker[j] > data$Marker[i]) &&

data$Status[j] == 1) {

sens <- sens + 1

} else if ((data$Marker[j] <= data$Marker[i]) &&

data$Status[j] == 0) {

spec = spec + 1

}

}

sens <- sens/ncases

spec <- spec/ncontr

rocmatr[i+1,1] <- sens

rocmatr[i+1,2] <- 1-spec

if (i < nsam) {

rocmatr[i+1,3] <- (data$Marker[i+1] + data$Marker[i]) /2

} else {

rocmatr[i+1,3] <- data$Marker[i]

}

}

class(rocmatr) <- c("roctab", "matrix")

return(rocmatr)

}

rocauc <- function(rocmatrix) {

# Estimate of AUC by the trapezoidal rule

auc <- 0.0

n <- length(rocmatrix[,1])

for (i in 2 : n) {

auc <- auc + 0.5 * (rocmatrix[i-1,1] + rocmatrix[i,1]) *

(rocmatrix[i-1,2] - rocmatrix[i,2])

}

return(unname(auc))

}

rocseauc <- function(rocmatr, marker, status) {

# Estimate of AUC, its standard error and the corresponding p value,

# using the method by DeLong et al, 1988

auc <- rocauc(rocmatr)

# Temporary dataframe, sorted and with valid data only

Data <- data.frame(Marker = marker, Status = status)

Data <- na.omit(Data)

Data <- Data[order(Data$Marker),]

nsam <- length(Data$Status)

ncases <- sum(Data$Status)

ncontr <- nsam - ncases

V10 <- array(0, dim=ncases)

V01 <- array(0, dim=ncontr)

S10 <- 0.0

S01 <- 0.0

IV10 <- 0

IV01 <- 0

for(i in 1:nsam) {

if(Data$Status[i] == 1) {

IV10 <- IV10 + 1

} else if (Data$Status[i] == 0) {

IV01 <- IV01 + 1

}

for (j in 1:nsam) {

if (Data$Status[i] == 1 && Data$Status[j] == 0) {

if (Data$Marker[i] > Data$Marker[j]) {

V10[IV10] = V10[IV10] + 1.0/ncontr

} else if (Data$Marker[i] == Data$Marker[j]) {

V10[IV10] = V10[IV10] + 0.5/ncontr

}

} else if (Data$Status[i] == 0 && Data$Status[j] == 1) {

if (Data$Marker[i] < Data$Marker[j]) {

V01[IV01] = V01[IV01] + 1.0/ncases

} else if (Data$Marker[i] == Data$Marker[j]) {

V01[IV01] = V01[IV01] + 0.5/ncases

}

}

}

}

for (i in 1:ncases) {

S10 = S10 + (V10[i] - auc)^2 / (ncases - 1)

}

for (i in 1:ncontr) {

S01 = S01 + (V01[i] - auc)^2 / (ncontr - 1)

}

se = sqrt(S10/ncases + S01/ncontr)

zauc = abs(auc - 0.5) / se

pval = 2*(1 - pnorm(zauc))

CI95L <- max(0,auc-1.96*se)

CI95U <- min(1,auc+1.96*se)

output <- c(auc, se, CI95L, CI95U, pval)

names(output) <- c("AUC", "SE(AUC)","95%CI L", "95%CI U", "p")

return(output)

}

rocaucfromdor <- function(dor) {

# Estimate of AUC in a proper ROC from DOR

if (dor == 1) {

return(0.5)

} else {

return(dor/(dor-1)-dor*log(dor)/(dor-1)^2)

}

}

rocdor <- function(auc, eps = 0.0001, MAXITER = 1000L) {

# Estimate of DOR under a proper model assumption

MAXDOR <- 100000 # Maximum value admitted for DOR

eauc <- 0.5 # Estimated AUC

incr <- 1.0 # Increment for DOR estimate

if (auc < 0.5) {

print("The curve is not proper (AUC < 0.5)")

return(-1)

} else if (auc > 1) {

print("AUC cannot be > 1.0")

return(-1)

} else if (auc == 0.5000) {

return(1)

} else if (auc == 1.0000) {

print("DOR is infinite. DOR = 10000 is returned")

return(10000)

} else if (auc > 0.9999) {

return(MAXDOR)

}

# Early approximated estimate of DOR

# using a Taylor series expansion

if (auc < 0.6137) { # DOR range: about 1-2

dor <- 29 - sqrt(16.3 - 25.4*auc)

incr <- 0.5

} else if (auc < 0.7172) { # DOR range: about 2-4

dor <- 27.8 - 20*sqrt(3.2 - 2.5*auc)

incr <- 1.0

} else if (auc < 0.8268) { # DOR range: about 4-10

dor <- 12.2 - 25*sqrt(0.83 - auc)

incr <- 3.0

} else if (auc < 0.8867) { # DOR range: about 10-20

dor <- 179.6 * auc - 139

incr <- 5.0

} else if (auc < 0.9286) { # DOR range: about 20-40

dor <- 515 * auc - 438

incr <- 10.0

} else if (auc < 0.9464) { # DOR range: about 40-60

dor <- 1159 * auc - 1038

incr <- 10.0

} else if (auc < 0.9631) { # DOR range: about 60-100

dor <- 2504 * auc - 2314

incr <- 20.0

} else if (auc < 0.9764) { # DOR range: about 100 - 180

dor <- 6420 * auc - 6090

incr <- 40.0

} else if (auc < 0.9818) { # DOR range: about 180 - 250

dor <- 13947 * auc - 13445

incr <- 35.0

} else { # DOR > 250

dor <- 530 # AUC = 0.9900

incr <- 50

}

# Refining DOR estimate by a "divide and conquer" approach

niter <- 0L

eauc <- rocaucfromdor(dor)

delta <- auc - eauc

direc <- sign(delta) # Direction for iteration (-1 for decrement)

while((abs(delta) > eps) || (niter == MAXITER)){

niter <- niter + 1

dor <- dor +direc*incr

if (dor < 1.0) {

dor <- 1.0

incr <- incr/2

direc <- +1 # Summing increment

}

# Control for DOR < 1.0 here

eauc <- rocaucfromdor(dor)

if (sign(auc - eauc) != direc) {

incr <- incr/2

direc <- -direc

}

delta = auc - eauc

}

return(dor)

}

biasproproc <- function(dor) {

if(dor < 1.0) {

# The ROC curve is not proper

return(-1)

}

if(dor == 1.0) {

return(0.0)

}

bias <- ((dor - 1) * sqrt(dor) - dor * log(dor)) / ((dor - 1)^2)

return(bias)

}

plotroc <- function(roctable1, roctable2 = NULL, roctable3 = NULL) {

# Display ROC curves on a ROC plot

plot.new()

sens1 <- roctable1[,1]

onespec1 <- roctable1[,2]

if (!is.null(roctable2)) {

sens2 <- roctable2[,1]

onespec2 <- roctable2[,2]

}

if (!is.null(roctable3)) {

sens3 <- roctable3[,1]

onespec3 <- roctable3[,2]

}

plot(onespec1, sens1, type = "o", col = "red",

ylab = "Sensitivity", xlab = "1-Specificity")

abline(0,1, lty = 2)

if (!is.null(roctable2)) {

lines(onespec2, sens2, type = "o", col = "blue")

}

if (!is.null(roctable3)) {

lines(onespec3, sens3, type = "o", col = "green")

}

return(NULL)

}

plotproper <- function(dor, color = "black") {

# Add a theoretical binormal proper ROC curve to a ROC plot

datamatr <- proproc(dor)

onespec <- datamatr[,1]

sens <- datamatr[,2]

lines(onespec, sens, col = color)

return(NULL)

}

proproc <- function(dor, npoints = 2001L) {

# Calculate the coordinates of a theorical proper binormal ROC curve

if (dor < 0.5) {

print("ROC curve is improper")

return -1

}

x <- c(1:npoints) # 1 - Specificity

x <- (x-1)/npoints

x[npoints] = 1.0

y <- dor*x/(dor*x+1-x) # Sensitivity

datamatr <- matrix(c(x,y), nrow = npoints, ncol = 2)

return(datamatr)

}

######################################################################

# Example of application of the R routines used in this study

# for the analysis of ITT data set, described in the paragraph 6.2

ROCITT <- roctable(ITTData$ITT, ITTData$Status)

rocseauc(ROCITT, ITTData$ITT, ITTData$Status)

AUC SE(AUC) 95%CI L 95%CI U p

0.92204301 0.03442276 0.85457441 0.98951161 0.00000000

# Optimal cut-off (redundant output omitted):

ROCITT

Sens 1-Spec Cut-off

..... ........... ........... ...........

[46,] 0.83870968 0.16666667 -8.4499998

..... ........... ........... ...........

# The minus (“-“) sign has been added to the marker values to prevent the ROC

# curve from lying under the chance line, because lower concentrations were

# associated with a higher probability of disease

plotroc(ROCITT)

plotproper(rocdor(rocauc(ROCITT)))


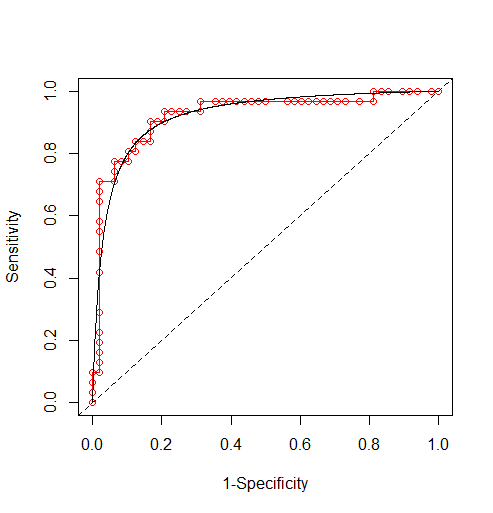


# Metz and Kronman test

library(pcvsuite)

ROC_Parm_ITT <- rocreg(dataset = "ITTData", d = "Status", markers = "ITT")

MKT(ROCITT, ROC_Parm_ITT)

[1] 0.6438119

# Estimate of the “bias” according to equation (8):

biasproproc(rocdor(rocauc(ROCITT)))

[1] 0.06632563
